# Antibiotic Receipt During Outpatient Visits for COVID-19 in the US, From 2020 to 2022

**DOI:** 10.1001/jamahealthforum.2022.5429

**Published:** 2023-02-17

**Authors:** Samuel R. Wittman, Judith M. Martin, Ateev Mehrotra, Kristin N. Ray

**Affiliations:** 1 Department of Pediatrics, University of Pittsburgh School of Medicine, University of Pittsburgh Medical Center Children’s Hospital of Pittsburgh, Pittsburgh, Pennsylvania; 2 Department of Health Care Policy, Harvard Medical School, Boston, Massachusetts

## Abstract

This cross-sectional study examines prescribed and filled antibiotics for outpatient COVID-19 treatment among children, adolescents, and adults with commercial insurance.

## Introduction

Approximately 30% of outpatient COVID-19–related visits among Medicare beneficiaries have resulted in a filled antibiotic prescription.^1^ We examined antibiotic receipt at COVID-19–related visits for commercially insured individuals aged 0 to 64 years.

## Methods

We identified outpatient visits with an *International Statistical Classification of Diseases and Related Health Problems, Tenth Revision* (*ICD-10*) diagnosis code of U07.1 between April 1, 2020, and May 31, 2022, by children and adolescents (≤17 years) and adults (18-64 years) in the OptumLabs Data Warehouse, which contains deidentified claims from 10% to 20% of commercially insured individuals in the US. The Harvard Institutional Review Board deemed this cross-sectional study exempt from review and waived the informed consent requirement because deidentified data were used. We followed the STROBE reporting guideline.

Consistent with prior research,^1^ we identified outpatient visits by individuals with both medical and pharmacy coverage and their association with antibiotic fills within 7 days before or after these visits (eMethods in Supplement 1). We limited analysis to COVID-19–related visits and excluded 5% of these visits (and associated antibiotics) with a codiagnosis for which antibiotics may be appropriate (eTable in Supplement 1).^2^ We classified all remaining visits as in-person physician office, practice-based telemedicine, direct-to-consumer telemedicine, emergency department (ED), urgent care, or other (eMethods in Supplement 1).^3^ Statistical analysis was performed with SAS 9.4 (SAS Institute).

## Results

**Figure 1.**
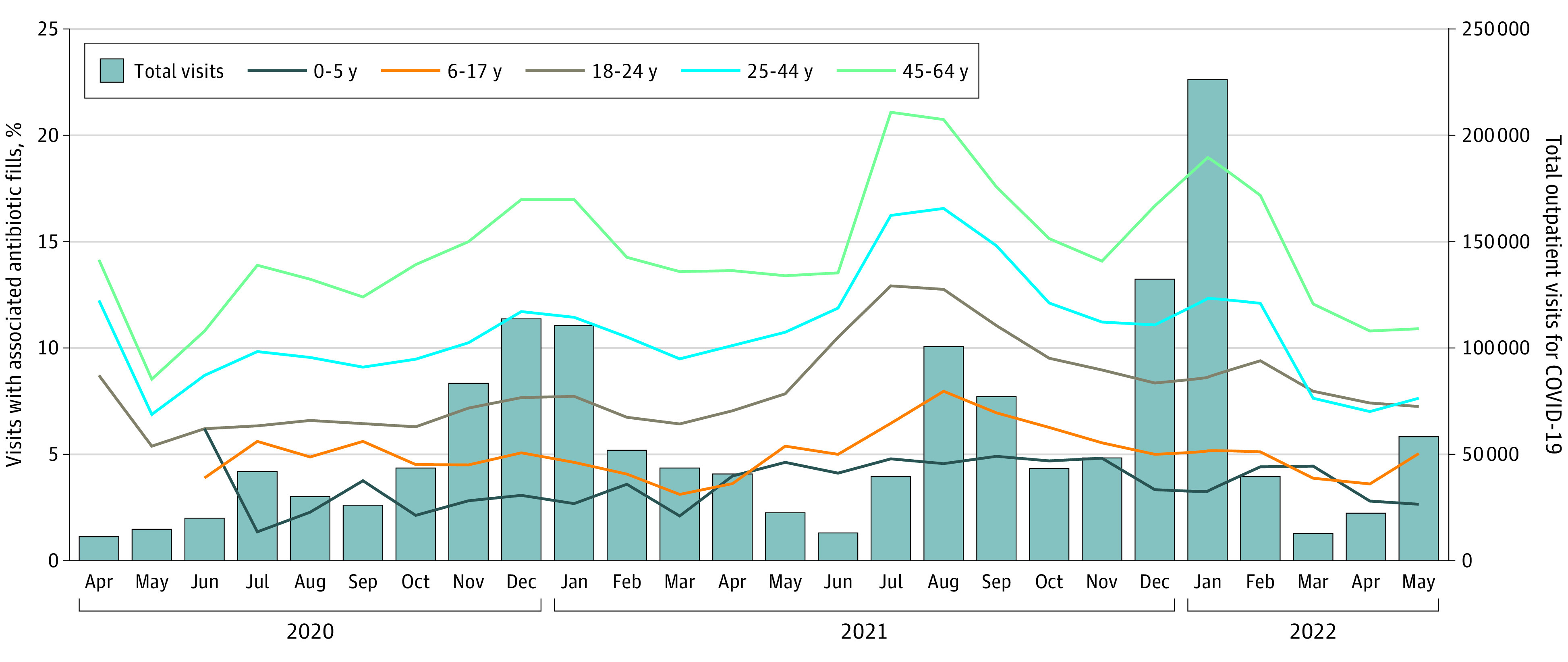
COVID-19 Outpatient Visits and Antibiotic Receipt by Age Group Data for 0-to-5-year and 6-to-17-year age groups were suppressed from April to May 2020 in compliance with cell size policy of the data source.

**Figure 2.**
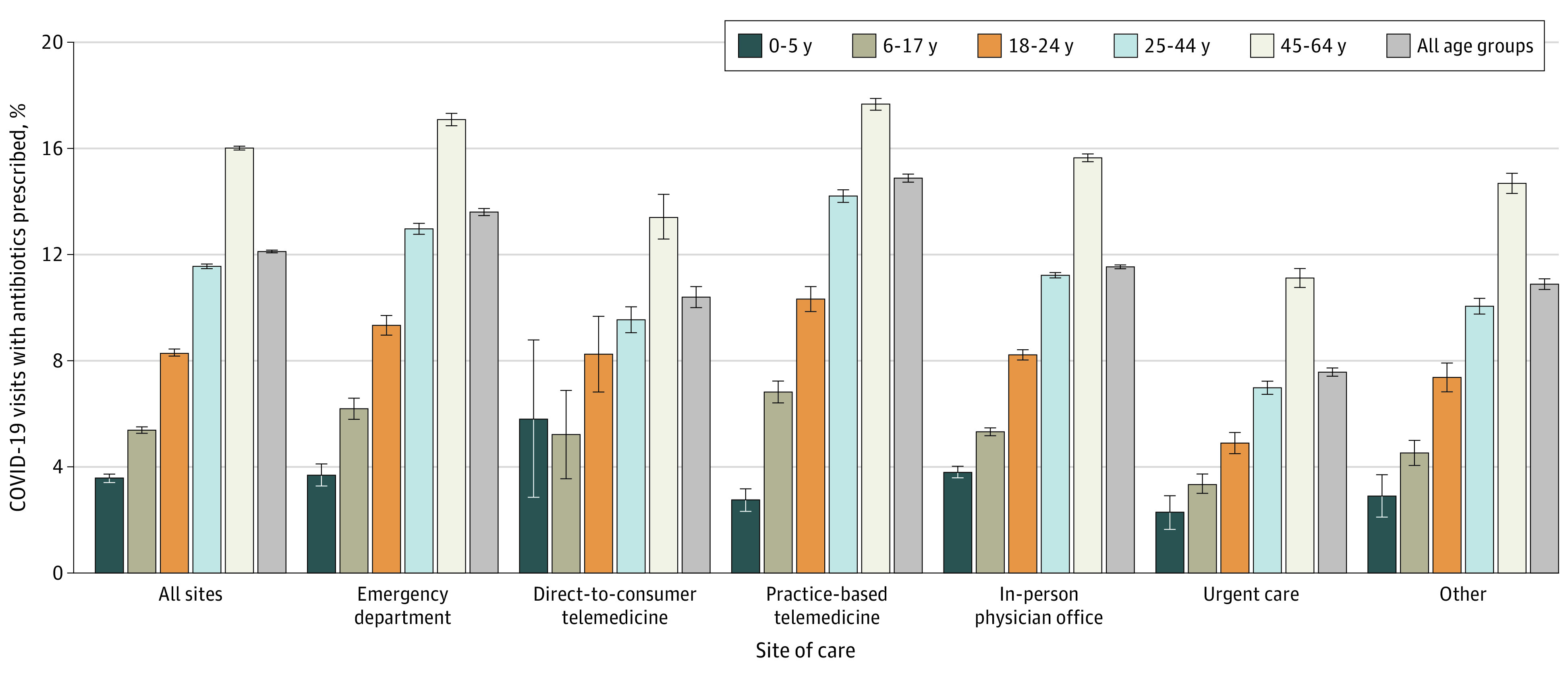
Antibiotic Receipt by Age Group and Site of Care The 95% CIs (error whiskers) for population proportions were calculated using the Wald interval for binomial proportions. The eMethods in Supplement 1 describe the sites of care. The “other” category included visits to nonphysician specialists and visits without taxonomy codes.

We included 1 293 303 adult visits and 177 057 children and adolescent visits. Antibiotic receipt during COVID-19–related visits increased with age: 4% in 0-to-5-year to 16% in 45-to-64-year age groups (Figure 1 and Figure 2). COVID-19–related visits accounted for 20% of acute respiratory tract infection (ARTI)–related visits and 7% of ARTI-related antibiotic receipt among all groups. In sensitivity analyses of antibiotic fills within 2 (vs 7) days of visits, antibiotic receipt was 4% vs 5% in children and adolescents and 11% vs 13% in adults.

COVID-19–related visits in children and adolescents vs adults occurred predominantly in physician offices (66% vs 51%), followed by EDs (12% vs 18%) and practice-based telemedicine (11% vs 17%). Antibiotic receipt varied by site of care, with highest rates at practice-based telemedicine and ED visits for all groups except 0 to 5 years (Figure 2). Among children aged 0 to 5 years, antibiotic receipt was highest during direct-to-consumer telemedicine visits. Antibiotic receipt was highest in the South (15%), followed by the West (9%), Midwest (9%), and Northeast (7%).

Among children younger than 6 years, the most common antibiotic was amoxicillin (37%) followed by azithromycin (36%). In those aged 6 to 17 years and adults, azithromycin (68% and 70%) was more commonly received than amoxicillin (15% and 4%).

## Discussion

Antibiotic receipt at COVID-19–related visits was substantially lower for children and adolescents than adults and varied by region and site of care. Increased prescriptions in adults may be associated with a higher prevalence of comorbidities and higher risks of adverse outcomes.

The findings that, among both adults and those aged 6 to 17 years, antibiotic receipt was highest at ED and practice-based telemedicine visits and that azithromycin was the most common were consistent with prior results.^1^ Azithromycin might be selected for its potential anti-inflammatory and antiviral properties, especially before more data were available.^4^ Amoxicillin use among the youngest group suggests concerns about associated (but undiagnosed) bacterial infection such as otitis media or pneumonia.

Study limitations included the lack of data on Medicaid-covered encounters or visits by uninsured individuals. Additionally, data were collected when pediatric visits and antibiotic use for non–COVID-19 ARTI-related visits were lower than usual.^5^ The *ICD-10* code U07.1 appears highly specific but not as sensitive for COVID-19–related visits.^6^ Claims data did not include prescribed but not filled antibiotics or antibiotics purchased without insurance. We also did not examine how severity of illness or presence of comorbidities was associated with antibiotic use. Understanding these prescribing practices will inform efforts to improve antibiotic stewardship.

## Notes

Supplement 1.
eMethods
**eTable.** Diagnosis for Which Antibiotics May Be Appropriate

Supplement 2.
Data Sharing Statement
