# Supplementary material for: Antibiotic Receipt During Outpatient Visits for COVID-19 in the US, From 2020 to 2022
Source: JAMA Health Forum. 2023 Feb 17;4(2):e225429. doi: 10.1001/jamahealthforum.2022.5429 (PMC9938423; doi:10.1001/jamahealthforum.2022.5429)
Supplement: Supplement 1. — eMethods eTable. Diagnosis for Which Antibiotics May Be Appropriate [file jamahealthforum-e225429-s001.pdf]

Supplementary Online Content

Wittman SR, Martin JM, Mehrotra A, Ray KN. Antibiotic receipt during outpatient visits for COVID-19 in the US, from 2020 to 2022. *JAMA Health Forum.* 2023;4(2):e225429. doi:10.1001/jamahealthforum.2022.5429

eMethods

eTable. Diagnosis for Which Antibiotics May Be Appropriate

This supplementary material has been provided by the authors to give readers additional information about their work.

© 2023 Wittman SR et al. *JAMA Health Forum.*

eMethods

Identifying visits and linking antibiotics: We identified 2.9 million children <18 and 10.6 million adults 18-64 who were enrolled with both medical and pharmacy coverage during April 2020-May 2021. This population includes enrollees in every US state. We identified all visits with a diagnosis for COVID-19 (U07.1) or one of the antibiotic-appropriate diagnoses listed in Supplemental Table 1. We filtered visits so that each member had at most one visit per day, prioritizing visits with antibiotic-appropriate diagnoses. We then linked each antibiotic fill in the data to a single visit within 7 days before or after the fill; if multiple visits occurred during that +/- 7-day window, we first prioritized any visit with an antibiotic appropriate diagnosis, and then the visit that was temporally closest to the fill. For our analysis focused on COVID-19 visits, we then excluded all visits with antibiotic-appropriate diagnoses (and the associated linked fills). Finally, we excluded all visits that did not have an outpatient CPT code (99201-99499), leaving only COVID-19 outpatient visits and the linked antibiotic fills.

Classifying site-of-care: ED visits were identified by the presence of one of the following: revenue codes 0450-0452, 0459, or 0981; place of service code 23; CPT codes 99281-99285; or a provider taxonomy code for Emergency Medicine. Among remaining visits, UC visits were identified by: revenue code 0456; place of service code 20; or a provider taxonomy code for Urgent Care. Among remaining visits, office visits were identified by a provider taxonomy code consistent with primary care (such as Family Practice, Pediatrician, Physician Assistant, or Nurse Practitioner) or with physician specialists (such as Cardiologist, Otolaryngologist, etc.). Telemedicine visits were identified by: CPT modifiers GT, GQ, or 95; place of service code 2; or CPT codes 99441-99445. Telemedicine providers were classified based on volume and visit type: providers with at least 25 visits per year and >80% telemedicine by volume were classified as DTC telemedicine providers, while other providers with telemedicine visits were classified as practice-based telemedicine providers. Telemedicine visits were classified as DTC vs. practice-based according to the provider classification. The category of "Other" included visits to non-physician specialists (such as Psychologist, Optician, etc.) and visits that did not include taxonomy codes.

Sensitivity analysis: We performed a sensitivity analysis where we shortened the window to link visits with antibiotic fills from 7 days to 2 days.

© 2023 Wittman SR et al. *JAMA Health Forum.*

eTable. Diagnosis for which antibiotics may be appropriate

Supplemental Table 1<br>Diagnosis for which antibiotics may be appropriate		
**Diagnosis**	**Included Conditions**	**ICD-10 Codes**
Acute otitis media	Acute serous, allergic, and nonsuppurative otitis media; chronic serous, mucoid, allergic, and nonsuppurative otitis media	H65.0n, H65.11n, H65.19n, H65.2n, H65.3n, H65.41n, H65.49n, H65.9n, H66.00n, H66.01n, H66.1n, H66.2n, H66.3Xn, H66.4n, H66.9n, and/or H67.n
Pharyngitis	Acute pharyngitis and tonsillitis	J02, J02.n, J03, J03.00, J03.8n, and/or J03.9n
Sinusitis	Acute maxillary, frontal, ethmoidal, sphenoidal, and pansinusitis	J01.0n, J01.1n, J01.2n, J01.3n, J01.4n, J01.8n, and/or J01.9n
Additional bacterial infections	Central venous catheter infections, infection after immunization, bites and scratches, infections due to specific pathogens (eg, Salmonella), sexually transmitted diseases, neutropenia, cellulitis, abscess, otitis externa, chronic otitis media, pneumonia, chronic sinusitis, impetigo, lymphangitis, lymphadenitis, acne and other skin conditions, septic arthritis, osteomyelitis and discitis, myositis, pyelonephritis, cystitis, urinary tract infection, salpingitis, peritonitis, pelvic infections, sepsis, and bacteremia	T80.2n, T88.0n, W50.3n, W53.n1Xn, W54.0XXn, W55.n1Xn, W56.n1Xn, W58.n1Xn, W59.n1Xn, W61.n1xn, Y04.1xxn, A01.0n, A01.n, A02.n, A03.n, A04.n, A18.0n, A22.7, A23.n, A26-27.n, A28.0, A28.2-9, A32.n, A33, A37.n, A38.1-9, A40-41.n, A42.7, A48-59.n, A63.8, A64, A69.2n, A74.8-9n, B37.7, B78.1, B95.n, B96.n, D70.n, H00.036, H05.01n, H60.0-3n, H62.4n, H66.1-3n, I00, I01.n, J13-8.n, J20.0-2, J32.n, J36, J39.0-1, K04.0, K04.4, K04.6-7, K12.2, K61.n, L01-8.n except L03.213 and L08.1, L70.n, L73.0, L88, L92.8, L98.0, L98.3, M00-1.n, M46.2-3n, M60.0n, M86.n, N11.0-1, 13N.6, N15.1, N15.9, N16, N30.n, N34.0-2, N39.0, N70.n, N71-7.n, N98.0, P36-9.n, R65.n, and/ or R78.81

Adapted from Kronman MP, Gerber JS, Grundmeier RW, et al. Reducing antibiotic prescribing in primary care for respiratory illness. Pediatrics. 2020;146(3):e20200038. doi:10.1542/peds.2020-0038.

© 2023 Wittman SR et al. *JAMA Health Forum.*
