# Supplementary material for: Antibiotic Receipt During Outpatient Visits for COVID-19 in the US, From 2020 to 2022
Source: JAMA Health Forum. 2023 Feb 17;4(2):e225429. doi: 10.1001/jamahealthforum.2022.5429 (PMC9938423; doi:10.1001/jamahealthforum.2022.5429)
Supplement: Supplement 2. — Data Sharing Statement [file jamahealthforum-e225429-s002.pdf]

Data Sharing Statement

Wittman. Antibiotic Receipt During Outpatient Visits for COVID-19 in the US, From 2020 to 2022. *JAMA Health Forum*. Published February 17, 2023.
doi:10.1001/jamahealthforum.2022.5429

Data
Data available: No
